# Spotted wolffish (*Anarhichas minor*) sperm cryopreservation in 5-mL cryovials

**DOI:** 10.1007/s10695-020-00837-1

**Published:** 2020-07-01

**Authors:** J. Beirão, S. Flengstad, I. Babiak

**Affiliations:** Faculty of Biosciences and Aquaculture, Nord University, NO-8049 Bodo, Norway

**Keywords:** Spotted wolffish, Sperm cryopreservation, Cryotubes, Sperm motility

## Abstract

In spotted wolffish *Anarhichas minor* aquaculture, cryopreservation is used to secure sperm availability throughout the entire spawning season. Under current protocols, sperm is cryopreserved in 0.5-mL straws. This implies thawing a considerable number of straws for insemination with cryopreserved sperm. In this work, we scale up the spotted wolffish sperm cryopreservation procedure through the development of a protocol for sperm cryopreservation in 5-mL cryovials. Different freezing (distances from the liquid nitrogen surface) and thawing rates were tested. The best results were obtained with cryovials frozen at a distance of 1.5 cm from the liquid nitrogen surface and thawed either at 15 or 10 °C for 4 and 6 min, respectively. Under these conditions, similar percentage of motile cells, sperm velocity and percentage of viable cells were obtained in comparison with the sperm cryopreserved in the traditional 0.5-mL straws. This protocol will facilitate the process of insemination with cryopreserved sperm in the spotted wolffish hatcheries.

## Introduction

Farmed spotted wolffish (*Anarhichas minor*) males do not show normal spawning behaviour and, as a consequence, females release unfertilized eggs. Thus, gametes have to be stripped and fertilization conducted in vitro (Le François and Archer 2007; Beirão and Ottesen 2018). Spotted wolffish sperm is unique compared with sperm of other farmed species, because it is already motile at stripping, remains constantly motile for up to 2 days and loses its motility upon a contact with seawater (Kime and Tveiten 2002; González-López et al. 2020). In this species, cryopreservation is used to secure sperm availability when eggs are spawned, because it is often impossible to find spermiating males (Le François et al. 2008; Gunnarsson et al. 2009; Santana et al. 2020). Spotted wolffish spawn approximately 8000 to 30,000 large benthonic eggs (Falk-Petersen et al. 1999; Falk-Petersen and Hansen 2003). Currently, sperm is cryopreserved in 0.5-mL straws. In order to achieve good fertilization rates, depending on the sperm concentration, an approximate volume of 2 to 15 mL of fresh sperm and 2 h contact time (Beirão and Ottesen 2018) or 4 to 30 mL of cryopreserved sperm and 4 h contact time (Santana et al. 2020) is recommended to inseminate 1 L of eggs (≈ 5000 eggs). This implies thawing a considerable number of straws for insemination with cryopreserved sperm. Large cryopreservation volumes, such as 5- or 10-mL cryovials and macrotubes, have already been tested in several species (Babiak et al. 2008; Beirão et al. 2011; Riesco et al. 2017; Nomura et al. 2018; Herranz-Jusdado et al. 2019). Cryopreservation in large volumes makes it possible to store higher volumes of sperm, which is essential to inseminate large volumes of eggs (Cabrita et al. 2001). In order to scale up the spotted wolffish sperm cryopreservation procedure and facilitate handling of cryopreserved sperm, in this study, we developed a protocol for sperm cryopreservation in 5-mL cryovials.

## Material and methods

### Sample collection

Sperm samples were collected from spotted wolffish males at the fish farm AMINOR AS (Halsa, Nordland, Norway) on June 2018. The breeders were kept all-year-round in 1600-L rectangular tanks with an open flow through system under natural temperature conditions and photoperiod adjusted to mature in the spring. The fish was anaesthetised with 500 ppm tricaine methanesulfonate (MS-222, Sigma-Aldrich) for 5 min. Subsequently, the sperm collection procedure followed Beirão and Ottesen (2018). Briefly, to decrease the chance of the samples being contaminated with urine or faeces, the abdominal area was massaged to release the urine. Afterward, the stripping was done by pressing down on the lateral area of the abdomen where the gonads were located. The sperm was collected using Pasteur pipettes. Only sperm samples with a motility (%) above 50% were used, and the sperm from at least three different males was pooled each time in order to have enough volume to conduct the experiment. Fresh samples were kept at 4 °C and analysed within 1–3 h after the first individual sperm sample was collected.

### Sperm quality evaluation

Sperm motility parameters were measured in 4 μL of pre-diluted sperm (3 μL of sperm in 17 μL of the solution developed for spotted wolffish by Kime and Tveiten (2002): 145 mM NaCl, 4.55 mM CaCl_2_, 4.83 mM KHCO_3_, 2.37 mM MgSO_4_, and 1 mM glucose; pH 7.5 and 310 mOsm/kg with the addition of 1% bovine serum albumin (Sigma-Aldrich), hereafter KT solution). The percentage of motile sperm and curvilinear velocity (VCL) were evaluated with a CASA system (Computer Assisted Sperm Analysis) SCA 6.2–Motility module (Microptic, Barcelona, Spain). The system was attached to a microscope (Nikon Eclipse Ci, Tokyo, Japan) with a camera (Basler acA1300-200uc, Ahrensburg, Germany). The microscope stage temperature was kept at 6 °C with a stage temperature controller (Linkam T95-PE, Tadworth, United Kingdom). The CASA system was adjusted to 20 frames/s, 1 s acquisition time, 10–50 μm^2^ for head area. Cells that were moving slower than 9 μm/s were considered drifting. The CASA video was set to record after the drift had stopped, between 1 and 2 min, and each sample was analysed in triplicate.

Sperm viability, or the percentage of cells with intact plasma membrane, was measured using a commercial kit (LIVE/DEAD™ Sperm Viability Kit, Invitrogen). A SYBR-14 working solution was prepared by diluting 2 μL of SYBR-14 1 mM stock solution (already in aliquots) with 18 μL of the KT solution resulting in a concentration of 100 nM. Sperm was diluted 1:10 in an Eppendorf tube to a volume of 200 μL in the KT solution. A portion 0.5 μL of the 100 nM SYBR-14 working solution was added to the diluted sperm and the suspension incubated in the dark for 10 min at 4 °C. After the incubation, 1.5 μL of PI 2.4 mM stock solution was added to the Eppendorf tube and incubated for 5 min more. After the incubation, 2 μL of the cell suspension was pipetted onto a microscope slide and the number of green and red-stained cells was counted in a fluorescent microscope. At least 100 cells were counted per slide, and three slides were analysed per sample. The percentage of viable cells was calculated.

### Cryopreservation trial

The sperm was diluted 1:1 with the KT solution containing 10% DMSO and 5% bovine serum albumin and loaded into 5-mL cryotubes. A 2-min equilibration on ice was applied prior the freezing. Cryotubes were then placed on a floating rack at either 1.5 or 4.5 cm distance from the liquid nitrogen (LN_2_) surface inside a styrofoam box (30 × 36 cm). After 10 min, the cryotubes were placed directly into the LN_2_. The cryotubes were thawed in a water bath at either 10 °C for 6 min (for subsamples cryopreserved at 1.5 and 4.5 cm distance from LN_2_) or 15 °C for 4 min (only for samples cryopreserved at the 1.5 cm distance from LN_2_). The thawing time was the shortest that the samples took to completely liquefy. The freezing and thawing paces were defined in a preliminary trial. A control subsample was cryopreserved in 0.5-mL straws at 4.5 cm distance from LN_2_ and thawed at 5 °C for 1 min according to the protocol by Santana et al. (2020). The cryotubes and control straws were stored in a LN_2_ storage tank until thawing for analysis. The cryotubes and straws used for sperm motility analysis were thawed the following day, the cryotubes and straws used for sperm viability analysis were transported in a LN_2_ dry shipper to our laboratories in Bodø and analysed after 1 month. Four different pools were used to evaluate the sperm motility parameters in fresh and cryopreserved sperm. Two different pools were used to evaluate sperm viability in cryopreserved sperm.

### Statistical analysis

All the data were tested for normality using a one-sample Kolmogorov-Smirnov test and homogeneity of variance by Bartlett’s test before being analysed with a one-way ANOVA using R statistical software 3.5.3. When the ANOVA showed that there was a significant difference, a post hoc Tukey test was used to specify the differences between treatments. For the VCL (μm/s), the Bartlett test indicated that there was no homogeneity of variances (*p* = 0.0003672), most likely because of the variability observed for the sperm cryopreserved in cryotubes using a freezing height of 4.5 cm. A Kruskal-Wallis test was decided to be used instead on the dataset for VCL. In all tests, a significant level (*α*) 0.05 was considered. No statistical analysis was conducted for the viability due to insufficient data (*n* = 2).

## Results and discussion

**Fig. 1:**
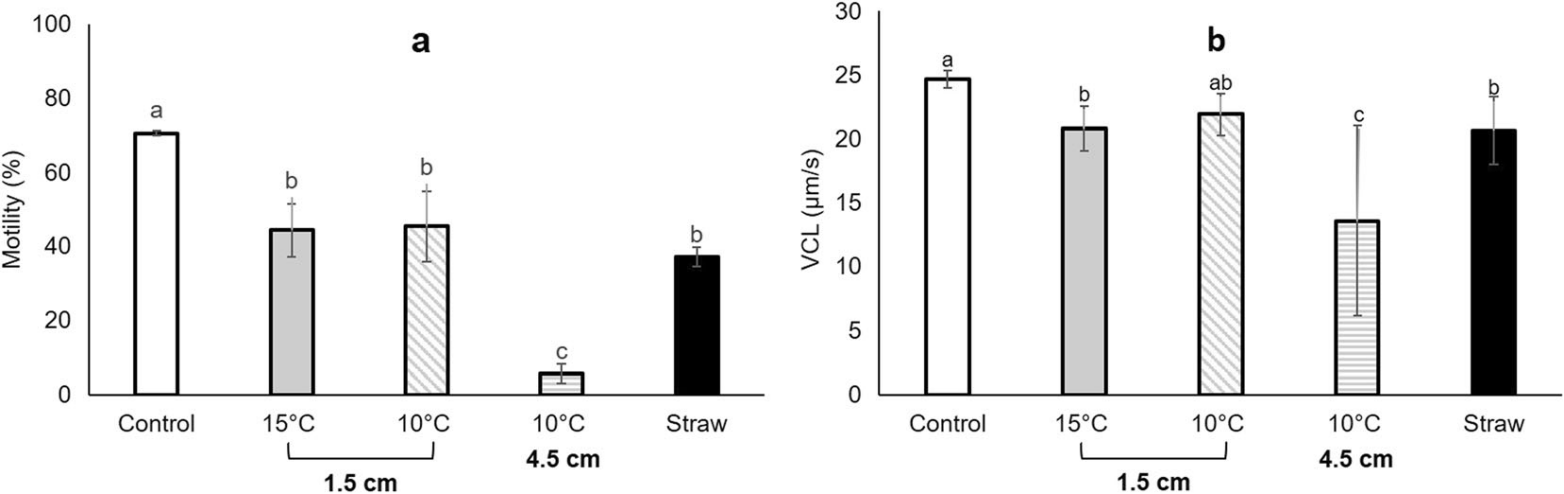
**a** Percentage of sperm motility and **b** curvilinear velocity (VCL) in the different treatments. The white bar represents fresh sperm and the black bar represents sperm cryopreserved in 0.5-mL straws. For the subsamples cryopreserved in the 5-mL cryovials (grey bars), the different freezing heights from the LN_2_ are indicated in the bottom line of *X*-axis and the different thawing rates (thawing temperature) are indicated in the top line of the *X*-axis. Different letters stand for significant differences between treatments (*p* < 0.05). Values represent mean ± SEM (*n* = 4)

The spotted wolffish sperm could be preserved in large storage volumes without a decrease in quality compared with small volumes. The ANOVA for the percentage of motile cells revealed that there were significant differences between treatments (*F*_4,15_ = 39.4, *P* < 0.001; Fig. 1a). Lower percentages of motile cells were observed in all cryopreserved treatments compared with the fresh subsample. A significant difference in sperm velocity between the sperm cryopreserved in cryotubes using a freezing height of 4.5 cm and the fresh control was found (Fig. 1b). The lowest percentage of motile cells and sperm velocity were observed in samples cryopreserved at 4.5 cm distance from the LN_2_. For both sperm motility parameters (percentage of motile cells and sperm velocity), there was no difference between subsamples cryopreserved in the straws and in the cryotubes at 1.5 cm distance from the LN_2_, regardless of the thawing rate. Most authors report a decrease in sperm quality after cryopreservation using large volumes compared with 0.5-mL or 0.25-mL straws (Babiak et al. 2008; Beirão et al. 2011; Riesco et al. 2017); however, this loss is largely compensated by the possibility to store higher volumes of sperm that enable to inseminate large volumes of eggs (Cabrita et al. 2001). This decrease in quality is related to the larger diameter of the freezing container, which produces heterogeneous freezing and thawing rates (Riesco et al. 2017). Nonetheless, other authors have already established protocols where sperm quality was similar after cryopreservation in small and large volumes. As an example, Herranz-Jusdado et al. (2019) in European eel *Anguilla anguilla*, Velasco-Santamaría et al. (2006) in yamú *Brycon amazonicus* and Nomura et al. (2018) in Japanese eel *Anguilla japonica* did not observe significant differences between sperm cryopreserved in 0.5 mL (0.25 mL in case of Japanese eel) and 5 mL volumes in terms of motility. Also, Gunnarsson et al. (2009) evaluated cryopreservation of spotted wolffish sperm using 0.5-mL and 1.0-mL straws and found no significant difference in motility; however, in their study, the motility was evaluated subjectively and without controlling the microscope stage temperature. Sperm motility in the spotted wolffish ceases at 20 °C (Kime and Tveiten 2002) and we observed in our lab that it was significantly reduced at temperatures above 10 °C (data not shown).

**Fig. 2:**
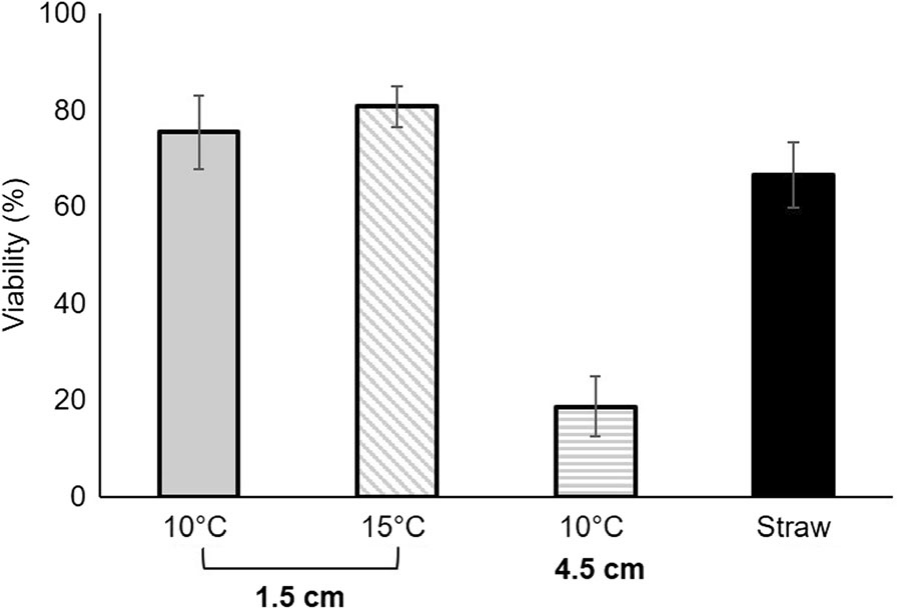
Percentage of sperm viability in the different treatments obtained with the dual staining SYBR-14/PI. The black bar represents the sperm cryopreserved in 0.5-mL straws. For the subsamples cryopreserved in the 5-mL cryovials (grey bars), the different freezing heights from the LN_2_ are indicated in the bottom line of *X*-axis and the different thawing rates (thawing temperature) are indicated in the top line of the *X*-axis. Values represent mean ± SEM (*n* = 2)

A similar trend was observed for the viability (Fig. 2) as in the case of the percentage of motile cells and sperm velocity. Viability of the sperm samples cryopreserved using cryotubes at freezing height of 1.5 cm and thawed at either 15 °C or 10 °C was similar to the viability of the samples cryopreserved in the 0.5-mL straws. While the sperm samples cryopreserved at freezing height of 4.5 cm had much lower viability. Cabrita et al. (2001) and Herranz-Jusdado et al. (2019) obtained similar sperm viability using 0.5 mL and 5 mL volumes for sperm cryopreservation in rainbow trout *Oncorhynchus mykiss* and European eel, respectively.

Usually, an adjustment in the freezing rates, as we did in our work, is needed to use large cryopreservation volumes (Cabrita et al. 2001; Beirão et al. 2011; Nomura et al. 2018). A lower distance from the LN_2_ for the cryovials was needed compared with the straws (1.5 cm vs 4.5 cm, respectively) in order to obtain the same post-thawing sperm quality. In this work, we did not have the equipment to monitor the internal temperature; however, other works have shown that for the same distance from the LN_2_, the larger volumes result in slower freezing rates (Cabrita et al. 2001; Velasco-Santamaría et al. 2006; Horvath et al. 2007; Beirão et al. 2011). This slower freezing rate can cause a greater cellular dehydration that could result in structural damages for the cell (Benson et al. 2012). Thus, as in our case, usually shorter distances from the LN_2_ for the cryovials are needed compared with the straws to obtain similar freezing rates and similar post-thaw sperm quality results. Alternatively, a longer freezing time could be also tested as suggested by Bokor et al. (2010) in wels catfish *Silurus glanis*.

Sperm cryopreserved in the 5-mL cryovials at 1.5 cm from the LN_2_ and thawed either at 15 or 10 °C presented similar percentage of motile cells, sperm velocity and percentage of viable cells in comparison with the sperm cryopreserved in the traditional 0.5-mL straws. In a previous work by Santana et al. (2020), fertilizations rate above 80% were obtained with spotted wolffish sperm cryopreserved in 0.5-mL straws. We recommend for the cryovials the sperm to be thawed at 10 °C for 6 min since this temperature is closer to the species normal temperature range. This protocol will facilitate the logistics of using spotted wolffish cryopreserved sperm at the hatcheries by making the process of fertilizing a large number of eggs more cost-efficient and less time-consuming.
